# Phylogeography and conservation genetics of the endangered *Tugarinovia mongolica* (Asteraceae) from Inner Mongolia, Northwest China

**DOI:** 10.1371/journal.pone.0211696

**Published:** 2019-02-07

**Authors:** Yanfen Zhao, Borong Pan, Mingli Zhang

**Affiliations:** 1 Key Laboratory of Biogeography and Bioresource in Arid Land, Xinjiang Institute of Ecology and Geography, Chinese Academy of Sciences, Urumqi, China; 2 University of Chinese Academy of Sciences, Beijing, China; 3 Institute of Botany, Chinese Academy of Sciences, Beijing, China; National Cheng Kung University, TAIWAN

## Abstract

*Tugarinovia* (Family Asteraceae) is a monotypic genus. It’s sole species, *Tugarinovia mongolica* Iljin, is distributed in the northern part of Inner Mongolia, with one additional variety, *Tugarinovia mongolica* var *ovatifolia*, which is distributed in the southern part of Inner Mongolia. The species has a limited geographical range and declining populations. To understand the phylogeographic structure of *T*. *mongolica*, we sequenced two chloroplast DNA regions (*psb*A-*trn*H and *psb*K-*psb*I) from 219 individuals of 16 populations, and investigated the genetic variation and phylogeographic patterns of *T*. *mongolica*. The results identified a total of 17 (H1-H17) chloroplast haplotypes. There were no haplotypes shared between the northern (*T*. *mongolica*) and southern groups (*T*. *mongolica* var. *ovatifolia*), and they formed two distinct lineages. The regional split was also supported by AMOVA and BEAST analyses. AMOVA showed the main variation that occurred between the two geographic groups. The time of divergence of the two groups can be dated to the early Pleistocene epoch, when climate fluctuations most likely resulted in the allopatric divergence of *T*. *mongolica*. The formation of the desert blocked genetic flow and enhanced the divergence of the northern and southern groups. Our results indicate that the genetic differences between *T*. *mongolica* and *T*. *mongolica* var. *ovatifolia* are consistent with previously proposed morphological differences. We speculate that the dry, cold climate and the expansion of the desert during the Quaternary resulted in the currently observed distribution of extant populations of *T*. *mongolica*. In the northern group, the populations Chuanjinsumu, Wuliji and Yingen displayed the highest genetic diversity and should be given priority protection. The southern group showed a higher genetic drift (F_ST_ = 1, G_ST_ = 1), and the inbreeding load (H_S_ = 0) required protection for each population. Our results propose that the protection of *T*. *mongolica* should be implemented through *in situ* and *ex situ* conservation practices to increase the effective population size and genetic diversity.

## Introduction

In recent years, phylogeographic studies of the arid region of Northwest China have increased and mainly focus on the impact of the Quaternary climate fluctuations on species’ phylogeographic patterns [1–3]. An increasing number of studies have shown that the deserts have an impact on the genetic structure and phylogeographic pattern of species, causing the speciation and population differentiation of many desert species [1, 4–6]. Evidence from pollen records indicates that ice sheets did not appear in arid Northwest China during the Quaternary [7]. However, glacial and interglacial cycles affected the evolutionary processes of species in this region [1, 8–10], through allopatric divergence [2, 11], range fragmentation, and regional range expansion [12, 13]. Additionally, the uplift of the Tibetan Plateau and global Pleistocene cooling promoted the formation and subsequent evolution of the desert [14, 15]. Several previous studies have shown that the increased aridification and desert expansion led to the speciation, habitat fragmentation, and diversification of desert plant species, as well as the distribution of montane plants on both sides of the desert [1, 2, 4, 16]. In addition, the desert zone acted as a geographical barrier that hindered gene flow among populations, which led to high genetic diversity among the populations and low genetic diversity within populations in arid Northwest China [4, 5, 16, 17]. However, few researchers have investigated the effects of desert formation on the evolutionary process of regional species in this arid region.

The arid regions of western Inner Mongolia contain many endemic species, several of which are considered endangered [18–21]. Specifically, the Alxa-Helan Mountain Range is considered to be one of eight high-diversity areas in China [22, 23]. *Tugarinovia* is a monotypic genus (*Tugarinovia mongolica* Iljin) with one additional variety (*T*. *mongolica* var. *ovatifolia*). *T*. *mongolica*, which is a member of the China Species Red List [24], is endemic to the gravel slopes of Inner Mongolia [25]. It is a perennial low herb with a dioecious reproductive system that flowers and fruits between May and June [26]. *T*. *mongolica* var. *ovatifolia* shows differences in morphology and habitat [26–28]. The major morphological differences between the two varieties are leaf and inflorescence size [29]. Based on field observations and herbaria specimen records, we believe this genus possesses disjunct distributions on the two sides of the desert. We find that *T*. *mongolica* is mostly distributed in the northern regions of the Alxa Desert, whereas its variety *T*. *mongolica* var. *ovatifolia* only occurs in narrow swaths southeast of the Alxa Desert. Currently, the combination of narrow distribution and overgrazing has resulted in a rapid decline of the species. Previous studies of *T*. *mongolica* have concentrated on embryology, taxonomy, origin, migratory route, and distribution patterns [29–32] but, to our knowledge, there have been no discussions of intraspecific taxonomy, phylogeography, or any aspect of conservation genetics.

In this study, we sequenced two chloroplast DNA sequences (*psb*A-*trn*H and *psb*K-*psb*I) to investigate the phylogeographic pattern of 16 populations of the genus *Tugarinovia* throughout its distributional range. Our study had the following aims. First, determine whether intraspecific phenotype variations of the genus are consistent with genetic differentiation. Second, identify whether Quaternary climate fluctuations (such as aridification and desert formation) affect the differentiation of *Tugarinovia*. Third, based on the genetic diversity and genetic structure of *T*. *mongolica* and *T*. *mongolica* var. *ovatifolia* populations, propose effective protection measures for the species.

## Materials and methods

### Sample collection

**Fig 1:**
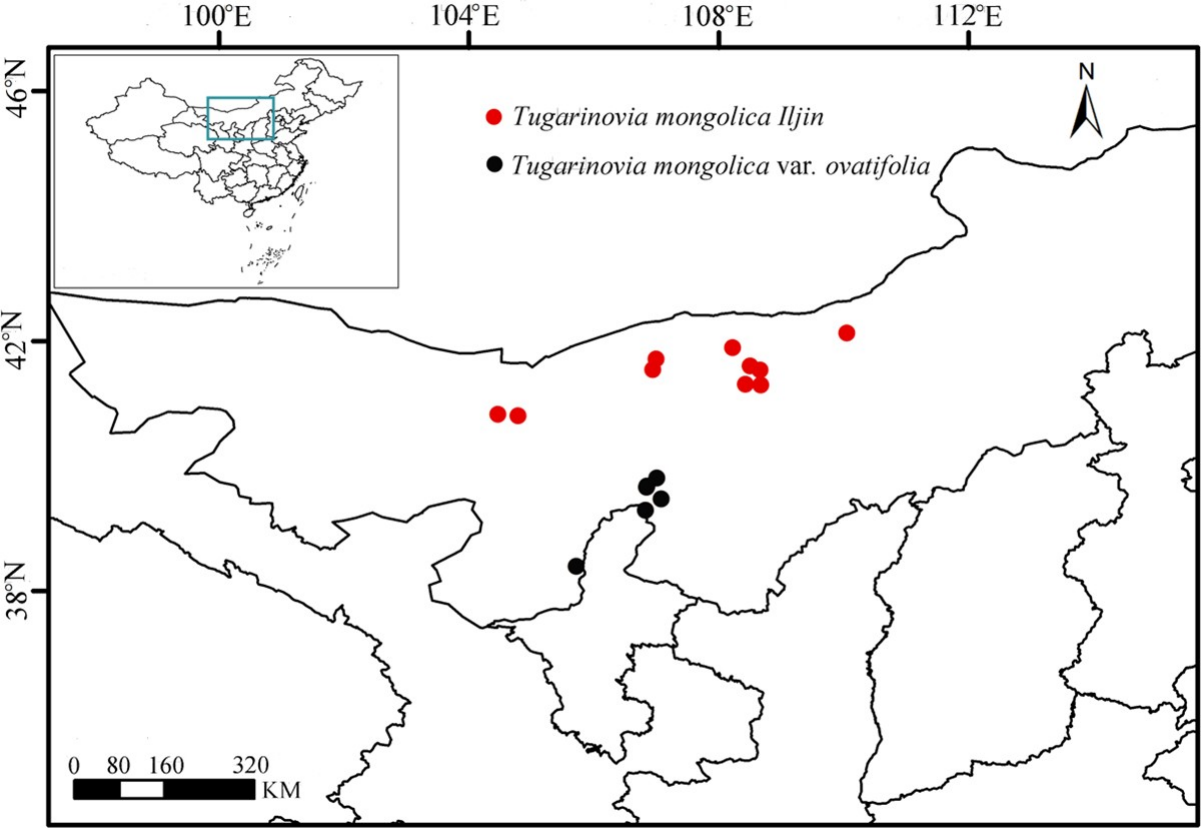
Study regions of *Tugarinovia mongolica*. Red dots represent *T*. *mongolica*, and black dots represent *T*. *mongolica* var. *ovatifolia*.

The study area was not a nature reserve and no specific permissions were required by the authoritative organization. Only one leaf was used as experimental material, so there was no serious damage to the target plant during field sampling. We collected leaf samples of 16 natural populations, which covered nearly the entire region occupied by *T*. *mongolica* from the northern part of the Alxa Desert to east of the southern part of the Alxa Desert, Inner Mongolia (Table 1, Fig 1). Ten populations belonging to *T*. *mongolica* (1–10) were sampled from northern Alxa, and six populations belonging to *T*. *mongolica* var. *ovatifolia* (11–16) from southeastern Alxa. We collected between 11 and 22 individuals from each population. Fresh leaves were sampled and dried immediately using silica gel in the field. Then, one sample of each population was deposited as a voucher specimen at the Herbarium of Xinjiang Institute of Ecology and Geography, Chinese Academy of Science (XJBI). We selected *Brachylaena huillensis* and *Atractylodes lancea* as outgroups in the phylogenetic analysis [31].

**Table 1:** Sample and genetic variation information for 16 populations of *Tugarinovia mongolica* in northwest China.

Species name	Population	Sample Location	Latitude/Longitude(N/E)	Altitude(m)	N	Haplotypes	Hd(±SD)	π(±SD)
	Overall				219	17	0.9086±0.0070	0.0092±0.0047
	NG	North Group			139	11	0.8250±0.0148	0.0065±0.0034
	1 HLT	Hailiutu,NM	41.60°/108.51°	1346	11	H1	0	0
	2 DLS	Delingshan,NM	41.29°/108.67°	1120	10	H1	0	0
	**3 CJSM**	**Chuanjinsumu,NM**	41.89°/108.22°	1336	12	H1,H2,H3	0.6818±0.0910	0.0055±0.0032
	4 BYH	Bayinhua,NM	42.13°/110.05°	1267	22	H4	0	0
***T*. *mongolica***	5 BYHT	Bayinhatai,NM	41.54°/108.66°	1341	16	H2	0	0
	6 SQ	Shuiquan,NM	41.31°/108.43°	1047	12	H1,H2	0.1667±0.1343	0.0015±0.0011
	7 SZ	Saizhen,NM	41.54°/106.95°	1582	15	H5	0	0
	8 BYT	Baoyintu,NM	41.71°/106.99°	1396	16	H1,H5,H6	0.5750±0.0799	0.0012±0.0009
	**9 WLJ**	**Wuliji,NM**	40.82°/104.47°	1448	11	H5,H7,H8	0.6909±0.0861	0.0041±0.0026
	**10 YG**	**Yingen,NM**	40.80°/104.79°	1338	14	H1,H5,H7,H8,H9,H10,H11	0.8462±0.0742	0.0131±0.0071
***T*. *mongolica var*. *ovatifolia***	SG	South Group			80	6	0.8408±0.0085	0.0045±0.0025
	11 LSM	Lashenmiao,NM	39.29°/106.83°	1134	11	H12	0	0
	12 DZT	Dizhentai,NM	39.68°/106.85°	1172	13	H13	0	0
	13 BRBL	Barunbieli,NM	38.39°/105.72°	1576	17	H14	0	0
	14 QPJ	Qipanjing,NM	39.47°/107.08°	1426	13	H15	0	0
	15 QLG	Qianligou,NM	39.80°/107.01°	1518	13	H16	0	0
	16 HBW	Haibowan,NM	39.65°/106.85°	1178	13	H17	0	0

Hd: haplotype diversity, π: nucleotide diversity. Bold letters indicate that the population has high genetic diversity.

### DNA extraction, PCR amplification, and sequencing

The total genomic DNA was extracted using a modified cetyltrimethy ammonium bromide (CTAB) protocol [33]. Initially, we screened ten pairs of chloroplast DNA regions (*trn*S-*trn*G, *psb*A-*trn*H, *psb*K-*psb*I, *rps*16-*trn*K, *rpl*32-*trn*L, *ycf*6-*psb*M, *trn*C-*rpo*B, *trn*V, *trn*D-*trn*T and *trn*L-*trn*F); however, only two plastid intergenic spacers (*psb*A-*trn*H and *psb*K-*psb*I) were found to be highly variable among individuals in the populations. Polymerase chain reaction (PCR) amplifications were performed in a total volume of 25 UL reactions with the amplification of two cpDNA regions, which were conducted by the following procedure: 95°C for 4 min, 36 cycles at 94°C for 30 s, 52°C for 30 s, 72°C for 1 min and, finally, 72°C for 10 min. PCR products were detected on 1.0% agarose gel and were purified using the QIAquick Gel Extraction Kit (Qiagen). These were then sequenced using an ABI Prism 3730 automated sequencer from Sangon Biotech Co., Ltd., Shanghai, China. Sequencing alignments were carried out in CLUSTAL W [34] and were both refined and adjusted manually. Finally, sequences representing all haplotypes were submitted to GenBank with accession numbers MK299501-MK299518.

### Genetic diversity and population structure

To understand the levels of genetic diversity of the species, haplotype diversity (Hd) and nucleotide diversity (π) for each population, the two geographic groups, and across all populations were calculated in ARLEQUIN 3.5 [35]. The total genetic diversity (H_T_), within-population genetic diversity (H_S_), and genetic differentiation (N_ST_, G_ST_) were estimated using the program Permut 1.0 with 1,000 permutation tests [36]. We used the parameters N_ST_ and G_ST_ to check whether a phylogeographic structure existed for all populations and the two geographic groups. Analysis of molecular variance (AMOVA) [37] was also performed to estimate the genetic structure by Arlequin 3.5, with significance tests based on 1,000 permutations [35]. The phylogenetic relationship among the haplotypes was constructed using Network v. 5.0 [38], followed by the median-joining (MJ) algorithm. Gaps were treated as a single mutation event. The spatial analysis of molecular variance (SAMOVA) was used to test the spatial genetic structure of cpDNA genetic variation using SAMOVA v. 1.0, where these analyses were performed for the range of 2 ≤ K ≤ 8 [39]. Finally, the best grouping was determined when the number of groups retained was maximized, F_CT_. However, this configuration was excluded when a single population appeared in the geographic group [40, 41].

### Population demographic analyses

To test whether the species experienced demographic expansions, the parameters of Tajima’s D and Fu’s F_S_ were estimated [42, 43]. A significant Tajima’s D value or a significant and large negative Fu’s F_S_ value indicated that the population had experienced demographic expansion. The sum of squared deviations (SSD) and raggedness index of Harpending (HRag) were also calculated [44]. At the same time, we calculated a mismatched distribution of pairwise differences [45]. The SSD value was measured using the *P*-value, for which a nonsignificant *P*-value and unimodal distribution of pairwise differences indicated that the population experienced recent expansion, whereas a significant *P*-value and multimodal mismatched distribution of pairwise differences indicated that the population did not experience recent expansion. Significant tests of the above analyses were estimated using Arlequin 3.5, with 1,000 permutation tests [35].

### Divergence time estimation

We estimated divergence times among the cpDNA haplotypes using BEAST v. 1.6.1 [46]. Since there were no fossil records, we used the reported substitution rate (1 and 3×10^−9^ s/s/y) based on the cpDNA substitution rates of most angiosperm species [47]. Based on the uncertainty of the rate values, we used a mean of 2×10^−9^ and an SD of 6.080×10^−10^ of the distribution to estimate the divergence times in this study [4, 48, 49]. We used the GTR substitution model and a coalescent tree prior. The Markov chain Monte Carlo (MCMC) permutations were run for 10,000,000 generations, sampling every 1,000 generations. The effective sample sizes (ESS) for the relevant estimated parameters were well above 200 by TRACER v. 1.5. We applied FigTree v. 1.3.1 to edit trees.

## Results

### Sequence analysis and haplotype distribution

**Fig 2:**
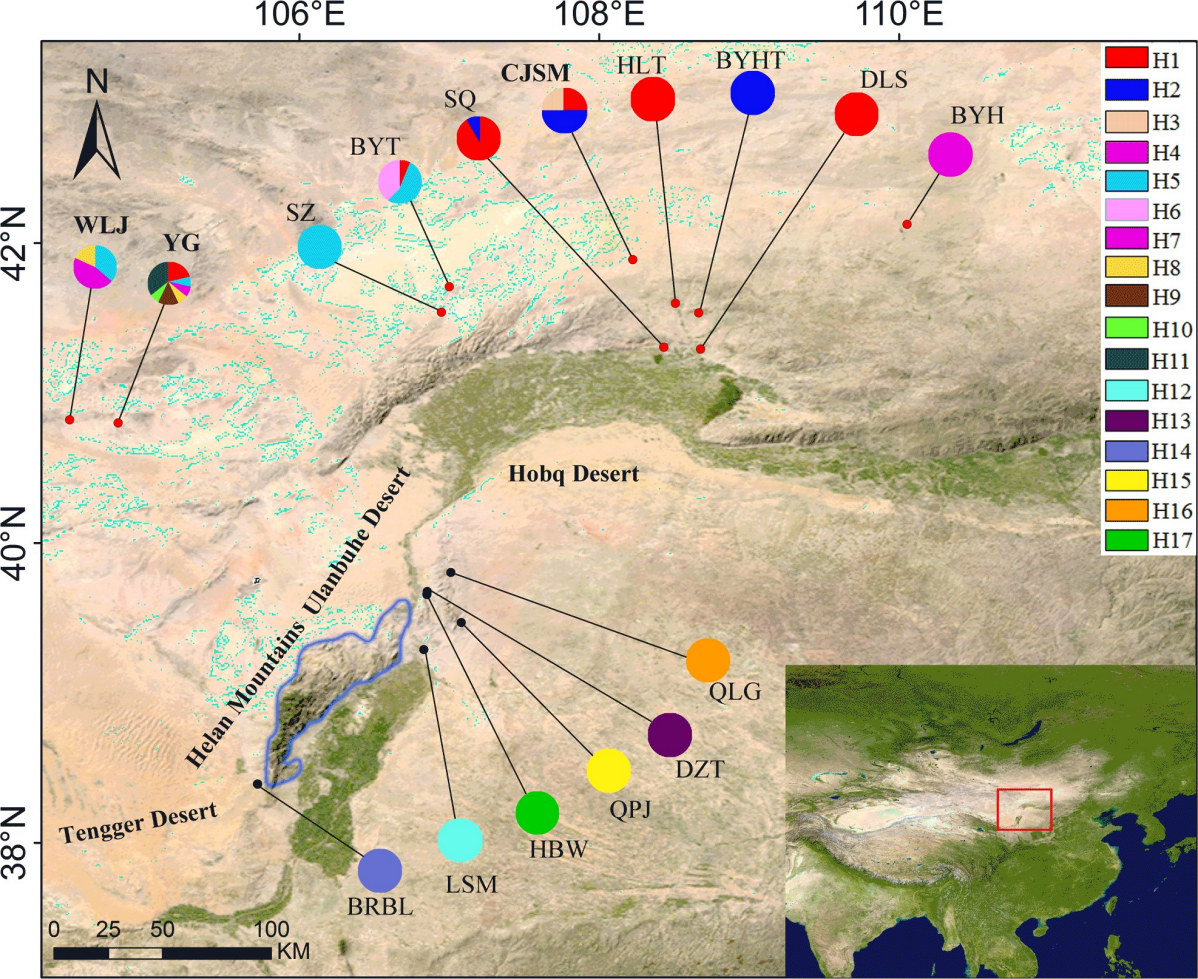
Geographic distribution of 17 cpDNA haplotypes detected in 16 populations of *Tugarinovia mongolica* from Inner Mongolia. Pie charts show the frequency of haplotype in each population. Red dots represent the population of the northern group (NG), and black dots represent the population of the southern group (SG). The blue line outlines represent the location of Helan Mountain. The nomenclature of NG and SG populations (See Table 1) is: HLT, Hailiutu; DLS, Delingshan; CJSM, Chuanjinsumu; BYH, Bayinhua; BYHT, Bayinhatai; SQ, Shuiquan; SZ, Saizhen; BYT, Bayintu; WLJ, Wuliji; YG, Yingen; LSM, Lashenmiao; DZT, Dizhentai; BRBL, Barunbieli; QPJ, Qipanjing; QLG, Qianligou; HBW, Haibowan.

**Fig 3:**
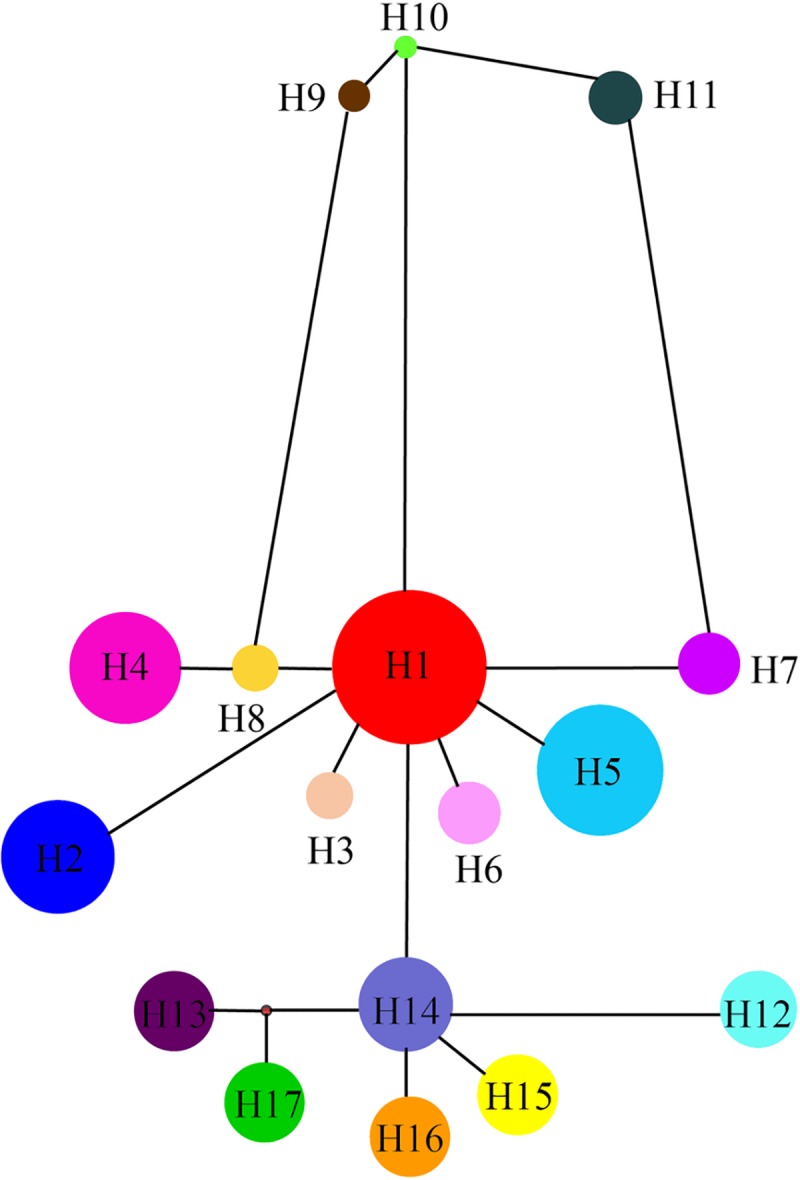
Statistical parsimony network of 17 haplotypes of *Tugarinovia mongolica* based on two cpDNA regions. The size of each circle is proportional to the frequency of the haplotype. The haplotypes H1-H11 are found exclusively in the northern group (NG), while haplotypes H12-H17 are found exclusively in the southern group (SG).

The sequences of *psb*A-*trn*H and *psb*K-*psb*I were both successfully amplified and sequenced in the 219 individuals from the 16 natural populations. The total length of the combined alignments was 897 bp. We were able to detect 21 variable sites, which included 16 substitutions and 5 indels (S1 Table). A total of 17 haplotypes were identified from all variable sites (Table 1). The results from the network analysis indicated that two clades, a northern group (NG) and southern group (SG) existed, with the northern group including 11 haplotypes (H1-H11) and the southern group including 6 haplotypes (H12-H17) (Figs 2 and 3). Importantly, no haplotype was shared between the two regions (Fig 3). In the northern group, haplotypes H1 and H5 were widespread, while haplotypes H3, H4, H6 and H9, H10, and H11 were fixed in the populations of CSJM, BYH, BYT and YG, and in the southern group, each of the 6 populations corresponded to a specific haplotype (Fig 2).

### Genetic diversity and population structure

The results of the SAMOVA also showed that as the number of groups increased to three, a single population emerged. We found that the optimal population grouping pattern of K = 2 was optimal: (1) populations 1–10 belonged to the northern group (*T*. *mongolica*), and (2) populations 11–16 belonged to the southern group (*T*. *mongolica* var. *ovatifolia*).

The total haplotype diversity (Hd = 0.9086±0.0070) was high, and the haplotype diversity of the southern group (Hd = 0.8408±0.0085) was slightly higher than that of the northern group (Hd = 0.8250±0.0148) (Table 1). The total nucleotide diversity (π) was 0.0092±0.0047, with the northern group (π = 0.0065±0.0034) exhibiting higher diversity than that of the southern group (π = 0.0045±0.0025) (Table 1). Throughout all populations of *T*. *mongolica*, the total genetic diversity, H_T,_ was 0.947 and the average within-population diversity, H_S,_ was 0.185. Although N_ST_ (0.841) was greater than G_ST_ (0.805), there was no significant difference between these two values (*P*>0.05). At the regional level, H_T_ and H_S_ were 0.858 and 0.296, and N_ST_ (0.623) was less than G_ST_ (0.655) for the northern group; for the southern group, the genetic diversity, H_T,_ was 1; the average within-population diversity, H_S,_ was 0; and N_ST_ (1) was equal to G_ST_ (1) (Table 2). We found no significant phylogeographical structures, either regionally or overall, in *T*. *mongolica*.

**Table 2:** Estimation of gene diversity (H_S_, H_T_) and gene differentiation (G_ST_, N_ST_) values of the total populations, the northern group (NG) and southern group (SG).

Region	N	H_S_	H_T_	G_ST_	N_ST_
**All region**	219	0.185(0.0782)	0.947(0.0290)	0.805(0.0840)	0.841(0.0747)
**NG**	139	0.296(0.1126)	0.858(0.0638)	0.655(0.1267)	0.623(0.1022)
**SG**	80	0	1	1	1

For the whole population, the results of AMOVA revealed that most of the total variance that occurred among the groups, among populations within the groups, and within the populations were small. A strong population genetic structure was detected in the species (F_ST_ = 0.88853, P<0.0001). In the northern group, variations among populations and within populations were 66.15% and 33.85%, respectively, whereas in the southern group, all variations were mainly presented among populations, with no variation within populations (F_ST_ = 1, P<0.0001) (Table 3).

**Table 3:** Results of analysis of molecular variance of cpDNA sequence data of *Tugarinovia mongolica*.

Source of variation	*d*.*f*.	*SS*	*VC*	*PV(%)*	Fixation index
**Among groups**	1	349.346	3.13894	52.86	F_CT_ = 0.52857**
**Among populations within groups**	14	416.909	2.13763	36	F_SC_ = 0.76356**
**Within populaitons**	203	134.375	0.66194	11.15	F_ST_ = 0.88853**
**Total**	218	900.630	5.93851		
**NG**					
**Among populations**	9	262.359	2.03571	66.15	
**Within populaitons**	129	134.375	1.04166	33.85	F_ST_ = 0.66151**
**Total**	138	396.734	3.07737		
**SG**					
**Among populations**	5	154.550	2.32668	100	
**Within populaitons**	74	0.000	0.00000	0	F_ST_ = 1.00000**
**Total**	79	154.550	2.32668		

Degrees of freedom (*d*.*f*.), sum of squares (*SS*), variance components (*VC*), percentage of variation (*PV*).

**, p<0.001, 1000 permutations.

NG: northern group, SG: southern group.

### Population demographic analyses

Demographic analysis of all populations and the two groups showed that the values of Fu’s F_S_ and Tajiam’s D were positive and not significant (S2 Table), which indicated that neither all populations nor the two groups experienced range expansion. We found further support from the mismatched distribution for all populations and for the two geographical groups, which were both multimodal (S1 Fig). Although the SSD value and raggedness index (P>0.05) showed a sudden expansion model, the results of Fu’s F_S_, Tajiam’s D and the mismatch analysis indicated that recent range expansion did not occur in *T*. *mongolica* (S2 Table, S1 Fig).

### Divergence time estimation

**Fig 4:**
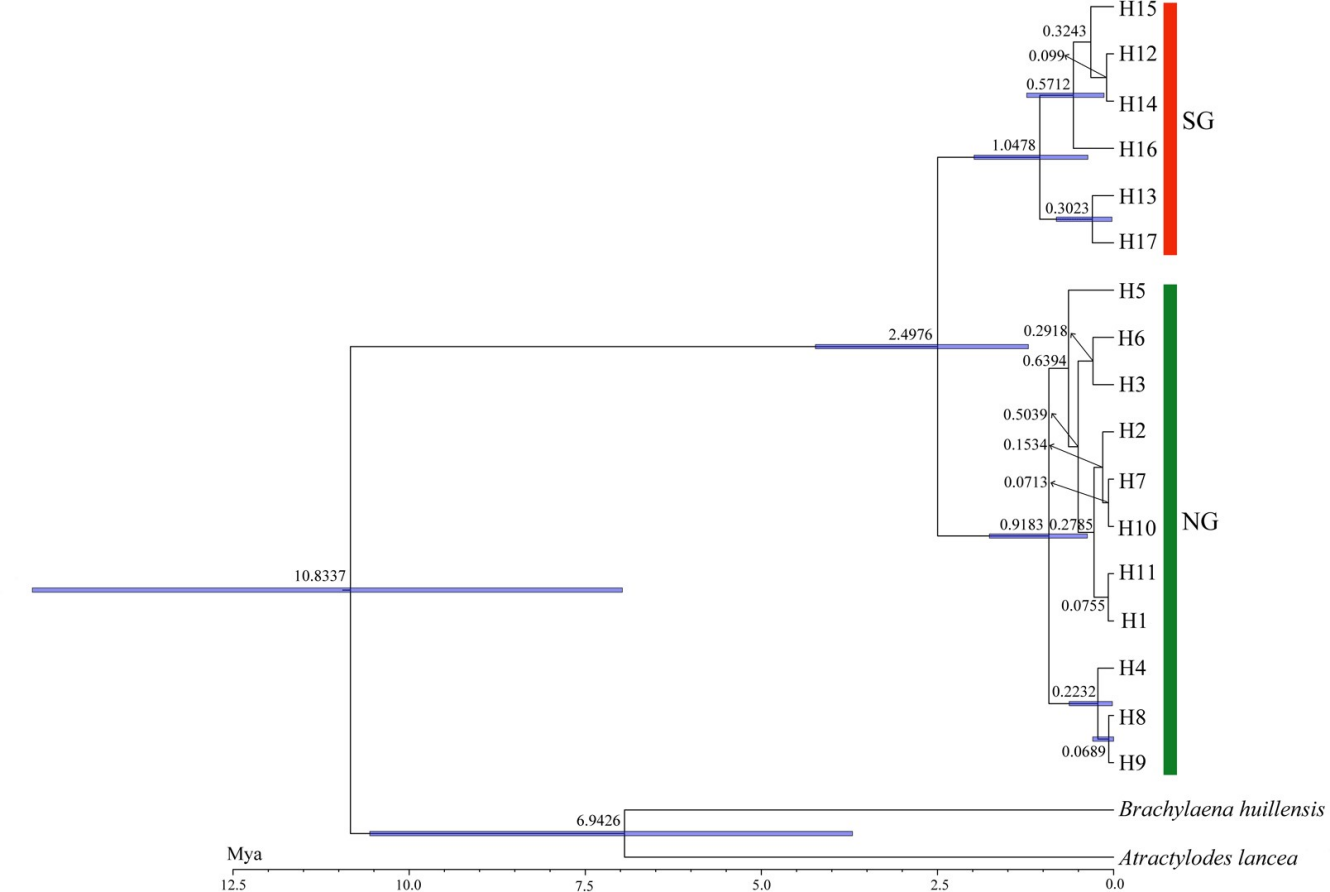
The divergence time (Mya) of 17 cpDNA haplotypes of the northern group (NG) and southern group (SG) of *Tugarinovia mongolica* based on BEAST analysis.

We found that the divergence time between the northern group (*T*. *mongolica*) and southern group (*T*. *mongolica* var. *ovatifolia*), which was determined from the BEAST analysis, occurred at 2.4976 (95%HPD: 1.2094–4.2318) Mya (Fig 4), during the early Pleistocene epoch.

## Discussion

### Allopatric divergence of *Tugarinovia mongolica* in Inner Mongolia

Based on the haplotype network and BEAST analysis of *T*. *mongolica*, there were two distinct clades that were clearly distributed in the northern and southern regions of Inner Mongolia (Figs 3 and 4). Furthermore, the AMOVA results showed that most of the genetic variation occurred between these two groups, along with a significant phylogeographic break that occurred between them. The SAMOVA showed the same results, divided into two groups, one northern group and one southern group. These results clearly indicate an allopatric divergence in *T*. *mongolica*. Several causes of this divergence could be: habitat fragmentation resulting from periodic oscillations of the Quaternary climate [50–52], lack of long distance dispersal [53], and geographic isolation [1, 4, 54, 55].

Based on morphological characteristics of *T*. *mongolica*, there are clear differences between *T*. *mongolica* and its variety *T*. *mongolica* var. *ovatifolia*. The leaves of *T*. *mongolica* are pinnately divided, long oval or rectangular, whereas the leaves of *T*. *mongolica* var. *ovatifolia* are ovate or oval, with a nondivided leaf margin and larger capitulum. We found evidence that *T*. *mongolica* var. *ovatifolia* in the southern group should be recognized as an independent species based on our molecular results, which is consistent with the morphological classification proposed by Zhao [29].

### High genetic differentiation due to vicariance

All populations of *T*. *mongolica* showed high levels of haplotype diversity (Hd = 0.9086) and low nucleotide diversity (π = 0.0092). Low nucleotide diversity is usually associated with a low seed amount and a small effective population size in some endangered species [18]. However, the low nucleotide diversity that was observed for *T*. *mongolica* may be attributed to the small effective population sizes that are associated with the reproductive barrier (male sterility). In general, species that have narrow distributions and small effective population sizes show high genetic differentiation among populations [17, 18], which we detected in this species (Table 2). *T*. *mongolica* showed strong genetic differentiation (G_ST_ = 0.805) and low genetic diversity within populations (H_S_ = 0.185). The above results indicated a high level of genetic differentiation among populations in *T*. *mongolica* that was due to restricted gene flow.

The divergence between the northern and southern groups can be traced back to the early Pleistocene epoch (Fig 4) during the development of arid conditions that led to the formation of the deserts that are located in Northwest China [15]. We speculate that the creation of the extreme climate may have resulted in the diversification of *T*. *mongolica*. In addition, the divergence time of two geographic groups (Fig 4) is consistent with the formation of the Hobq Desert [56, 57], which, as a geographical barrier, may have blocked the genetic flow between the northern and southern groups. Previous studies have shown that the desert, as a geographical barrier, promotes the allopatric divergence of species [4]. The pollination and fertilization requirements of *T*. *mongolica* make long-distance dispersal between the southern and northern groups impossible, because they were separated by deserts. Thus, we speculate that the desert may have acted as a geographic barrier that blocked gene flow between the two geographic groups, thereby resulting in allopatric divergence. Consequently, populations became isolated and independent in the northern and southern regions of the Alxa Desert.

In this study, each population of the southern group contained one specific haplotype, and most populations in the northern group shared one haplotype (Fig 2). The differentiation of haplotypes within the two groups occurred in the middle and late Pleistocene (Fig 4), and this period coincides with the formation period of the desert [14, 15]. The expansion of the desert may have led to habitat fragmentation [1]. Here, we use desert expansion to explain the fragmented distribution of *T*. *mongolica*. In addition, the biological characteristics (dioecious with male sterility and pistil abortion) of *T*. *mongolica* could have resulted in the distribution of extant populations. The fragmentation distribution of *T*. *mongolica* var. *ovatifolia* may also be related to desert expansion, but evidence of male sterility and pistil abortion in this group requires further research.

### Implications for *Tugarinovia mongolica* conservation

The results of genetic diversity and population structure are important to consider for the implementation of effective conservation strategies, particularly for endemic and endangered species [58, 59]. The risk of extinction is higher for species with narrow distributions and small population sizes, especially if the gene flow among populations is lower than those with large and stable populations. In addition, small population sizes are sensitive to reduced genetic diversity through genetic drift and inbreeding [60, 61].

According to the analysis of cpDNA data, the high genetic drift load (F_ST_ = 0.88853, G_ST_ = 0.805) and inbreeding load (H_S_ = 0.185) showed a significant extinction risk in the genus *Tugarinovia* (Table 2). This extinction risk is particularly noticeable in the populations of *T*. *mongolica* var. *ovatifolia*, which showed fragmented distributions, small population sizes, high genetic drift load (F_ST_ = 1, G_ST_ = 1), and high inbreeding load (H_S_ = 0) (Tables 2 and 3). All of the above indices can increase sensitivity to environmental changes and the risk of extinction. In addition, populations LSM, DZT, BRBL, QPJ, QLG and HBW of the southern group exhibited unique haplotypes, which offer some protection from extinction for the population of *T*. *mongolica* var. *ovatifolia*. In the northern group, the CJSM, WLJ and YG populations of *T*. *mongolica* exhibited higher genetic diversity than other populations. Since *T*. *mongolica* is a critically endangered, protected species with a second-class national priority [24], it is recommended that the hotspots of populations that contain the highest genetic diversity be protected [62, 63].

To mitigate genetic drift and the inbreeding load and increase the effective population size of *T*. *mongolica*, we propose establishing the following conservation measures. First, enact additional *in situ* conservation measures for the species, such as the creation of additional nature reserves in the northern and southeast Alxa Desert, especially the CJSM, WLJ, and YG populations of the northern areas. (The Wuhai location has established nature reserves for some endangered and rare species [19])’. In particular, nature reserves for *T*. *mongolica* var. *ovatifolia*, as an independent species with an unique haplotype, should be established. Second, a protocol should be developed for *ex situ* conservation actions, such as seed collection from natural populations and reproduction in botanical gardens or other places, which can ensure maximum conservation of the genetic diversity of species in those particular areas.

## Supporting information

S1 Table Variable sites of 17 Haplotypes (H1-H17) for *Tugarinovia mongolica* in the two cpDNA regions. (DOC)

S2 Table Parameters of mismatch distribution analyses—Tajima's D, Fu's Fs and mismatch distribution tests. (DOCX)

S3 Table The geographical coordinates of *Tugarinovia mongolica* from Inner Mongolia. (DOCX)

S1 Fig Mismatch distribution analysis of total populations, northern group (NG) and southern group (SG) based on two chloroplast DNA sequences data. (TIF)
