# Supplementary material for: Phylogeography and conservation genetics of the endangered Tugarinovia mongolica (Asteraceae) from Inner Mongolia, Northwest China
Source: PLoS One. 2019 Feb 7;14(2):e0211696. doi: 10.1371/journal.pone.0211696 (PMC6366884; doi:10.1371/journal.pone.0211696)
Supplement: S1 Table — (DOC) [file pone.0211696.s001.doc]

| Sequence position | | | | | | | | | | | | | | | | | | | | | |
| --- | --- | --- | --- | --- | --- | --- | --- | --- | --- | --- | --- | --- | --- | --- | --- | --- | --- | --- | --- | --- | --- |
|  | 13 | 15 | 114 | 248-255 | 276 | 316 | 324 | 331 | 340 | 373-380 | 381 | 419-434 | 540 | 590 | 680 | 704 | 714 | 734 | 739 | 747 | 815-819 |
| Haplotype | | | | | | | | | | | | | | | | | | | | | |
| H1 | C | C | A | - | T | T | C | C | A | - | T | - | A | G | A | T | G | G | T | C |  |
| H2 | C | C | A | - | T | T | C | C | A |  | T | - | A | G | A | T | G | G | T | C |  |
| H3 | C | C | A | - | T | T | C | C | A | - | T | - | G | G | A | T | G | G | T | C |  |
| H4 | C | C | A | - | T | T | T | C | A | - | T | - | A | G | A | T | G | A | T | C |  |
| H5 | C | C | A | - | T | T | C | C | A | - | T | - | A | T | A | T | G | G | T | C |  |
| H6 | T | C | A | - | T | T | C | C | A | - | T | - | A | G | A | T | G | G | T | C |  |
| H7 | C | C | A | - | T | T | C | C | A | - | T | - | A | G | A | T | G | G | T | C | - |
| H8 | C | C | A | - | T | T | C | C | A | - | T | - | A | G | A | T | G | A | T | C |  |
| H9 | C | C | A | - | T | T | C | C | A | - | T |  | A | G | A | T | G | A | T | C |  |
| H10 | C | C | A | - | T | T | C | C | A | - | T |  | A | G | A | T | G | G | T | C |  |
| H11 | C | C | A | - | T | T | C | C | A | - | T |  | A | G | A | T | G | G | T | C | - |
| H12 | C | C | G |  | T | G | C | C | A | - | - | - | A | G | A | C | A | G | C | C |  |
| H13 | C | C | G | - | G | G | C | A | A | - | - | - | A | G | G | C | A | G | C | C |  |
| H14 | C | C | G | - | T | G | C | C | A | - | - | - | A | G | A | C | A | G | C | C |  |
| H15 | C | A | G | - | T | G | C | C | A | - | - | - | A | G | A | C | A | G | C | C |  |
| H16 | C | C | G | - | T | G | C | C | C | - | - | - | A | G | A | C | A | G | C | C |  |
| H17 | C | C | G | - | G | G | C | A | A | - | - | - | A | G | A | C | A | G | C | A |  |

: ATCCTTTT; : ATATGTTC; :TGGAAAAATAAAATTA; :CAATT
