# Supplementary material for: Phylogeography and conservation genetics of the endangered Tugarinovia mongolica (Asteraceae) from Inner Mongolia, Northwest China
Source: PLoS One. 2019 Feb 7;14(2):e0211696. doi: 10.1371/journal.pone.0211696 (PMC6366884; doi:10.1371/journal.pone.0211696)
Supplement: S2 Table — (DOCX) [file pone.0211696.s002.docx]

| Population | Tajima’s D (*P* value) | Fu’s Fs (*P* value) | SSD (*P* value) | Hrag (*P* value) | Mismatch distribution |
| --- | --- | --- | --- | --- | --- |
| Overall | 1.1659(0.8820) | 5.8115(0.9150) | 0.0137(0.3670) | 0.0132(0.5600) | Multimodal |
| North group | 0.2989(0.6640) | 5.1871(0.9310) | 0.7602(0) | 0.0251(1) | Multimodal |
| South group | 1.5011(0.9280) | 5.6080(0.9910) | 0.0382(0.2950) | 0.0828(0.1930) | Multimodal |
