# Supplementary material for: Phylogeography and conservation genetics of the endangered Tugarinovia mongolica (Asteraceae) from Inner Mongolia, Northwest China
Source: PLoS One. 2019 Feb 7;14(2):e0211696. doi: 10.1371/journal.pone.0211696 (PMC6366884; doi:10.1371/journal.pone.0211696)
Supplement: S3 Table — (DOCX) [file pone.0211696.s003.docx]

|  | Population | Latitude/Longitude(N/E) | Altitude(m) |
| --- | --- | --- | --- |
| 1 | Hailiutu,NM | 41.60°/108.51° | 1346 |
| 2 | Delingshan,NM | 41.29°/108.67° | 1120 |
| 3 | Chuanjinsumu,NM | 41.89°/108.22° | 1336 |
| 4 | Bayinhua,NM | 42.13°/110.05° | 1267 |
| 5 | Bayinhatai,NM | 41.54°/108.66° | 1341 |
| 6 | Shuiquan,NM | 41.31°/108.43° | 1047 |
| 7 | Saizhen,NM | 41.54°/106.95° | 1582 |
| 8 | Baoyintu,NM | 41.71°/106.99° | 1396 |
| 9 | Wuliji,NM | 40.82°/104.47° | 1448 |
| 10 | Yingen,NM | 40.80°/104.79° | 1338 |
| 11 | Lashenmiao,NM | 39.29°/106.83° | 1134 |
| 12 | Dizhentai,NM | 39.68°/106.85° | 1172 |
| 13 | Barunbieli,NM | 38.39°/105.72° | 1576 |
| 14 | Qipanjing,NM | 39.47°/107.08° | 1426 |
| 15 | Qianligou,NM | 39.80°/107.01° | 1518 |
| 16 | Haibowan,NM | 39.65°/106.85° | 1178 |
