# Supplementary figures and images for: Phylogeography and conservation genetics of the endangered Tugarinovia mongolica (Asteraceae) from Inner Mongolia, Northwest China
Source: PLoS One. 2019 Feb 7;14(2):e0211696. doi: 10.1371/journal.pone.0211696 (PMC6366884; doi:10.1371/journal.pone.0211696)

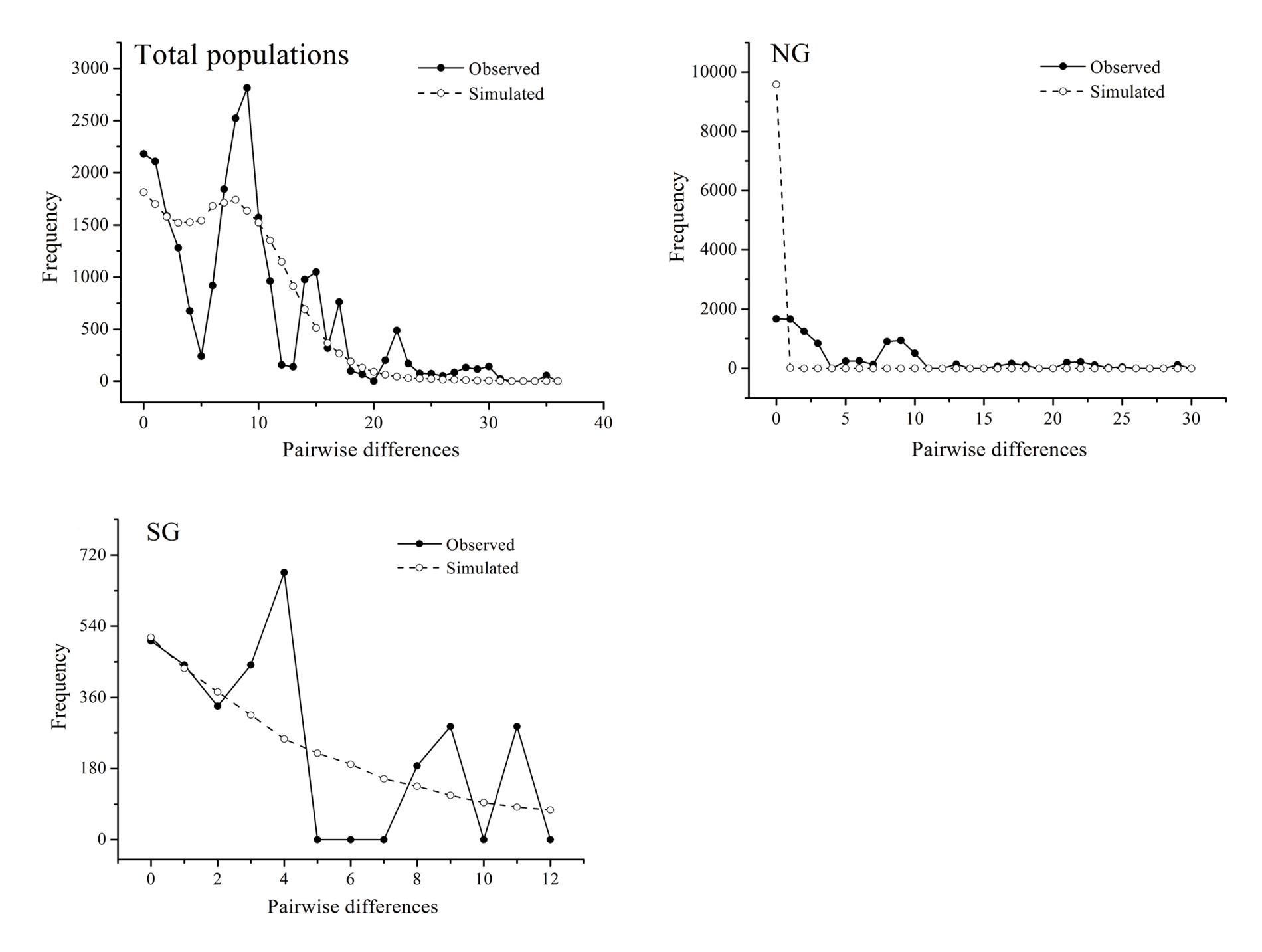

Supplement: S1 Fig — (TIF) [file pone.0211696.s004.tif]
